# Rare Biosphere Reveals a Decoupling Between Microbial Abundance and Intrinsic Physiological Potential in Shanxi Aged Vinegar Fermentation

**DOI:** 10.3390/foods15162942

**Published:** 2026-08-21

**Authors:** Yanfang Wu, Yan Li, Hanlin Chen, Xiuhong Zhang, Jia Song, Menglei Xia, Yu Zheng, Min Wang

**Affiliations:** 1 College of Food Science, Shanxi Normal University, Taiyuan 030092, China; 2 School of Biological Engineering, Tianjin University of Science and Technology, Tianjin 300457, China; 3 China Condiment Association, Beijing 100036, China

**Keywords:** Shanxi aged vinegar, meta-omics, abundance–physiological performance decoupling, rare biosphere, ecological constraint, *Pediococcus acidilactici*

## Abstract

The discrepancy between in situ microbial abundance and actual metabolic performance represents a critical challenge for interpreting microbial function from meta-omic data. Here, we integrated metagenomic and metatranscriptomic sequencing to investigate this decoupling between microbial abundance and cultivation-based physiological potential in Shanxi aged vinegar (SAV) solid-state fermentation. *Lactobacillus acetotolerans* dominated the community at both the genomic (40.89%) and transcriptomic (55.36%) levels, whereas *Pediococcus acidilactici* accounted for only 0.11%—a canonical rare-biosphere member. Source tracking via Sankey analysis showed that genes involved in acetate production were primarily attributed to *Acetobacter pasteurianus*, whereas genes involved in lactate production were predominantly associated with *Lactobacillus* spp. However, *L. acetotolerans* exhibited limited acid tolerance and lactic acid production, whereas the low-abundance *P. acidilactici* AAF1-5 displayed robust stress tolerance and superior lactic acid production under fermentation-relevant conditions—a striking contrast between microbial abundance and physiological performance. Metabolic interaction network analysis predicted that *P. acidilactici* may be co-inhibited by *L. acetotolerans* (*I_xy_* = −2.737, resource competition) and *A. pasteurianus* (*I_xy_* = −1.887, acid stress). To test whether ecological constraints, rather than intrinsic metabolic capacity, underlie this low abundance, we heterologously expressed the heat shock co-chaperone gene *grpE* from *A. pasteurianus* in *P. acidilactici* AAF1-5 as an experimental tool. The recombinant strain *P. acidilactici*-*grpE* exhibited significantly enhanced viability under acetic acid stress and, in simulated SAV fermentation, lactic acid content increased by 23.63% compared with the wild-type control. These results demonstrate that meta-omic abundance does not necessarily predict physiological performance and that low abundance may reflect ecological constraints rather than intrinsic functional deficiency. Our study provides an ecological framework for linking microbial abundance with physiological function beyond sequence-based abundance inference in complex fermentation microbiomes.

## 1. Introduction

Organic acids are the principal flavor determinants of cereal vinegar, with acetic acid providing the characteristic sharpness and lactic acid imparting mellowness and palate softness [1,2]. In traditional Chinese cereal vinegars produced by solid-state fermentation (SSF), lactic acid and acetic acid together account for over 85% of total organic acids [3,4], and their ratio critically determines the sensory balance between pungency and smoothness [5]. The formation of these organic acids is driven by the metabolic activities of the resident microbial community, in which *Lactobacillus* and *Acetobacter* are recognized as the core functional genera [4,6,7,8].

Shanxi aged vinegar (SAV) is produced through an open SSF system involving alcohol fermentation and acetic acid fermentation (AAF), followed by smoking and aging [9,10]. During AAF, microbes face extreme environmental stresses: temperatures up to 47 °C, ethanol exceeding 70 g/L, and acetic acid accumulating to >30 g/L [7,11,12]. The microbial community undergoes continuous succession driven by these gradients [7,13]. With the advent of high-throughput sequencing, metagenomics and metatranscriptomics have become routine approaches for profiling community composition and identifying functionally important microorganisms in fermented foods [14,15,16]. These methods have been widely applied to vinegar systems to identify core functional taxa and predict metabolic pathways [16,17,18,19]. Recently, attention has also been directed toward the ecological roles of low-abundance microorganisms—the “rare biosphere”—with studies showing that rare sub-communities can exhibit distinct succession patterns and that non-abundant species can function as keystone taxa in fermentation ecosystems [20,21,22,23,24].

Although these culture-independent approaches are powerful, a fundamental question remains: does meta-omic abundance accurately reflect physiological performance? Metagenomic sequencing quantifies the genetic potential of the entire community, while metatranscriptomics measures transcriptional activity at a given time point [14,15]. However, neither directly measures per-cell metabolic flux, enzymatic activity, or the rate of specific metabolite production [25,26,27]. Consequently, a species that is abundant at the DNA or RNA level may not be the primary functional contributor under process-relevant conditions. This potential discrepancy between ecological abundance and physiological performance represents a major obstacle to interpreting microbial function from meta-omic data alone. Addressing this question requires not only community profiling but also experimental approaches that can directly assess the intrinsic physiological capacity of individual community members.

Such a discrepancy was first suggested by our own previous work. In the course of our long-term studies of the SAV microbiome, we screened over 80 lactic acid bacteria (LAB) isolates from SAV and found that *Pediococcus acidilactici* AAF1-5 showed promising thermotolerance and lactic acid productivity, whereas *Lactobacillus acetotolerans*—the most abundant LAB species in the metagenome (40.89%)—exhibited limited culturability and limited acid tolerance [7,28,29]. These strain-level observations indicated a possible decoupling between ecological abundance and physiological performance. In parallel, our meta-omic analyses of the SAV community established that *Lactobacillus* and *Acetobacter* are the core functional genera and that genes involved in acetate and lactate metabolism can be tracked to specific taxa [5,8]. While these earlier studies provided valuable insights into the SAV community, they were mainly descriptive in scope: the quantitative comparison of genomic-versus-transcriptomic functional allocation across species, the quantification of ecological interactions constraining individual community members, and the experimental testing of whether the low abundance of a functionally potent rare biosphere member reflects ecological constraints rather than intrinsic metabolic deficiency remained to be addressed.

In this study, we integrated metagenomic and metatranscriptomic sequencing, metabolic interaction network analysis, and physiological characterization to (i) quantitatively compare the genomic-versus-transcriptomic allocation of organic acid metabolic functions across core species, (ii) construct a species-resolved metabolic interaction network to quantify the ecological constraints acting on *P. acidilactici*, and (iii) experimentally test the ecological constraint hypothesis by heterologous expression of the acid-resistance gene *grpE* from *Acetobacter pasteurianus* in *P. acidilactici* AAF1-5. We hypothesized that the low abundance of *P. acidilactici* in situ reflects ecological constraints rather than intrinsic metabolic deficiency. Among these constraints, acid stress was selected for experimental testing because it is the only one that can be relieved by a single, mechanistically defined genetic intervention, enabling an unambiguous causal test; resource competition, which arises from overlapping substrate use across multiple pathways, cannot be isolated by a single manipulation and was therefore left to future co-culture experiments.

## 2. Materials and Methods

### 2.1. Strains, Plasmids, and Media

*P. acidilactici* AAF1-5 (CGMCC 12062), *A. pasteurianus* AC2005 (CGMCC 3089) and *L. acetotolerans* CICC 10720 were previously isolated from vinegar fermentation. *Escherichia coli* MC1061 served as the cloning host. LAB were cultured in MRS medium (glucose 2%, peptone 1%, beef extract 1%, yeast extract 0.5%, sodium acetate 0.5%, Tween 80 0.1%, ammonium citrate 0.2%, K_2_HPO_4_ 0.2%, MgSO_4_·7H_2_O 0.058%, and MnSO_4_·4H_2_O 0.025%, pH 6.2–6.8). *A. pasteurianus* was cultured in GY medium (glucose 2%, yeast extract 2%, and ethanol 3.5% *v*/*v*). *E. coli* was grown in LB medium (1% tryptone, 0.5% yeast extract, and 1% NaCl, pH 7.2).

### 2.2. Sampling and Organic Acid Analysis

#### 2.2.1. Sampling

AAF samples (*Cupei*) of SAV were collected from Taiyuan, China. Samples on day 1, day 3, day 5, day 7, and day 9 represent the early stage, middle–early stage, middle stage, middle–late stage and late stage of fermentation, respectively. Samples were collected at a depth of 30 cm from the surface of *Cupei*. Samples for each day were collected from 3 parallel vats and within each vat a five-point sampling method (from five spatial positions) was used, following standard practice for heterogeneous SSF substrates. The samples from the three vats at each timepoint were then combined into a single representative sample for sequencing.

#### 2.2.2. Determination of Organic Acids

A high-performance liquid chromatography (HPLC) (Agilent, Santa Clara, CA, USA) system equipped with an Aminex HPX-87H 300 × 7.8 (mm) column (Bio-Rad, Hercules, CA, USA) was used for identification and analysis of organic acids. Organic acid standards (acetic acid, lactic acid, citric acid, malic acid, oxalic acid, pyruvic acid and tartaric acid; Sigma-Aldrich, St. Louis, MO, USA) were used for identification and quantification of organic acids in *Cupei*. A sample with 1.0 mL was diluted to 10.0 mL and centrifuged at 5000 rpm for 5 min, then filtered with a 0.45 μm microporous membrane. Mobile phase: 5 mmoL/L H_2_SO_4_; flow rate: 0.6 mL/min; injection volume: 20 μL; UV detector wave length: 215 nm; column temperature: 30 °C.

### 2.3. Metagenomic and Metatranscriptomic Sequencing and Analysis

Total DNA and RNA were co-extracted from *Cupei* samples using TRIzol-based sequential purification. Metagenomic libraries were prepared with the NEBNext Ultra DNA Library Prep Kit (New England Biolabs, Ipswich, MA, USA) and sequenced on the Illumina NovaSeq 6000 (Illumina, San Diego, CA, USA). Metatranscriptomic libraries were sequenced on the same platform. Reads were quality-filtered and assembled with Megahit v1.2.9. The taxonomic classification was performed using Kraken2 against the NCBI nt database, and functional annotation was conducted using the KEGG databases. Source tracking of organic acid synthesis genes was performed by mapping KEGG orthologs to assembled contigs and assigning taxonomy to each contig. Sankey diagrams were constructed to visualize the distribution of EC functional categories across core microbial species, comparing metagenomic coding potential with metatranscriptomic expression levels. Metagenomic and metatranscriptomic sequencing data from our earlier studies of the SAV microbiome [5,8] were further analyzed in the present study; they are available in the NCBI Sequence Read Archive under accession numbers PRJNA689964 and PRJNA602043, respectively. The present analysis extends the earlier work by integrated genome-versus-transcriptome source tracking, construction of a species-resolved metabolic interaction network, and quantitative comparison of genomic-versus-transcriptomic functional allocation.

### 2.4. Metabolic Interaction Network Analysis

The metabolic influence score *I_xy_* reflects the net metabolic interaction of species x on species y and was calculated following the framework of Sung et al. [30] as adapted in our previous study [31]. If species *x* exerts a greater growth-promoting than growth-inhibiting effect on species *y*, then *I_xy_* > 0, indicating a net positive (growth-promotive) interaction; conversely, if the growth-inhibiting effect predominates, then *I_xy_* < 0, indicating a net negative (growth-inhibitive) interaction. The score was calculated as follows:

$$I_{xy}=\frac{\partial \mu_{y}/\mu_{y}}{\partial n_{x}/n_{x}}\;=\;\frac{\;n_{x}}{\mu_{y}}\times \frac{\partial \mu_{y}}{\partial n_{x}}\;=\;\frac{\;n_{x}}{\mu_{y}}\sum_{m}\frac{\partial \mu_{y}}{\partial k_{my}}\frac{\partial k_{my}}{\partial n_{x}}$$

*n_x_*: Abundance of microbe *x*;

*μ_y_*: Change in growth rate or abundance of microbe *y*;

*k*: Each metabolite consumed by species *y*;

*k_my_*: Consumption rate of metabolite *k* per unit abundance of *y*.

To ensure edge reliability, the thresholds of the original method were applied: an abundance threshold *θ*_1_ = 0.001%, below which the contribution of a species to a metabolite term was set to zero; a net-influence threshold *θ*_2_ = 0.1, below which a link was discarded; and a minimum absolute influence *θ*_3_ = 10^−4^, below which links were removed as susceptible to noise. Based on the magnitude of *I_xy_*, interaction edges were classified as strong (|*I_xy_*| > 1.5), moderate (0.5 < |*I_xy_*| ≤ 1.5), or weak (|*I_xy_*| ≤ 0.5), where positive *I_xy_* values represent facilitative interactions and negative values represent inhibitory interactions. The network was visualized with Cytoscape v3.8.2.

### 2.5. Construction of Recombinant P. acidilactici Strains

#### 2.5.1. Gene Amplification and Vector Construction

Four acid-resistance-associated genes (*adhA*, *adhB*, *aatA*, and *grpE*) were selected a priori from *A. pasteurianus* AC2005 based on the established mechanisms of acetic acid resistance in acetic acid bacteria [32], with the aim of representing distinct, complementary resistance mechanisms: *adhA* and *adhB* (alcohol/aldehyde dehydrogenases) represent the ethanol-oxidizing respiratory chain supporting energy generation under high-acid conditions and are functionally redundant members of the same Adh family; *aatA* is associated with acetate assimilation/transport contributing to cytoplasmic acetate detoxification; and *grpE* is the nucleotide-exchange factor of the DnaK-DnaJ chaperone system involved in protein refolding and stress adaptation. Four acid-resistance-associated genes (*adhA*, *adhB*, *aatA*, and *grpE*) were amplified from *A. pasteurianus* AC2005 genomic DNA using the primers listed in Supplementary Table S1. The shuttle vector pNZ8148 was double-digested with KpnI and XbaI (37 °C, 50 min). Each gene was inserted using the ClonExpress II One Step Cloning Kit (Vazyme, Nanjing, China). Recombinant plasmids (pNZ8148-*adhA*, -*adhB*, -*aatA*, and -*grpE*) were transformed into *E. coli* MC1061 (42 °C, 90 s heat shock), selected on LB-chloramphenicol (25 μg/mL), and confirmed by colony PCR and Sanger sequencing (GENEWIZ, Suzhou, China).

#### 2.5.2. Construction of a Recombinant Expression Strain of *P. acidilactici* AAF1-5

Competent cells of *P. acidilactici* AAF1-5 were prepared as described [33]. Three microliters of each recombinant plasmid was electroporated (2.0 kV/cm, 5 ms; ECM 399, Bio-Rad). Cells were recovered in 800 μL MRS (37 °C, 2 h), then spread on MRS agar with 10 μg/mL chloramphenicol and incubated at 37 °C for 36–72 h. Transformants were confirmed by colony PCR and plasmid restriction analysis. Recombinant strains were designated *P. acidilactici*-*adhB*, -*aatA*, and -*grpE*; the empty vector control was *P. acidilactici*-pNZ8148. Gene expression in the recombinant strains was induced as follows: overnight cultures were diluted into fresh MRS medium and grown at 37 °C to the mid-exponential phase (OD600 ≈ 0.5); nisin was then added to a final concentration of 10 ng/mL, and the cultures were maintained under induction for 3 h before and during the subsequent experiments.

### 2.6. Simulated Solid-State Fermentation (SSSF) and Physicochemical Analysis

Prior to SSSF, the basic fermentation performance of the two strains was compared under laboratory liquid pure-culture conditions. Activated cultures of *P. acidilactici* AAF1-5 and *P. acidilactici*-*grpE* were inoculated into MRS liquid medium and incubated at 37 °C for 24 h. The fermentation performance of the two strains was then assessed by measuring pH, residual sugar, total acid, lactic acid, and acetic acid contents.

For the SSSF, activated cultures of *P. acidilactici* AAF1-5 and *P. acidilactici*-*grpE* were inoculated into MRS medium and incubated at 37 °C for 12 h. Cells were harvested by centrifugation at 4000 rpm for 5 min, washed twice with sterile saline, and resuspended in 10 mL of sterile saline. The cell suspension was inoculated into freshly collected *Cupei*, thoroughly mixed, and incubated at 30 °C. The *Cupei* was turned over once daily, and the contents of acetic acid and lactic acid were measured daily. For the determination of total acid and reducing sugar, samples were pretreated as follows: 5.0 g of *Cupei* was mixed with 45.0 mL of distilled water and incubated at 60 °C for 1 h. After cooling below 30 °C, 15.0 mL of the sample was centrifuged at 10,000 rpm for 10 min. Physicochemical indexes were determined according to previously reported methods [5,7]. Briefly, total acid was determined by titration with 0.1 M NaOH to the phenolphthalein endpoint, and reducing sugar was determined by the 3,5-dinitrosalicylic acid (DNS) colorimetric method using a glucose standard curve.

### 2.7. Statistical Analysis

All physiological and biochemical experiments were performed in triplicate. Data are expressed as the mean ± SD. One-way ANOVA with Tukey’s post hoc test was performed in SPSS v25.0 (IBM Corp., Armonk, NY, USA). *p* < 0.05 was considered significant. The metagenomic and metatranscriptomic data represent a single composite sample per timepoint, and the meta-omic comparisons are not based on formal hypothesis testing; no multiple-comparison correction was applied.

## 3. Results

### 3.1. Organic Acid Dynamics During SAV Acetic Acid Fermentation

**Figure 1:**
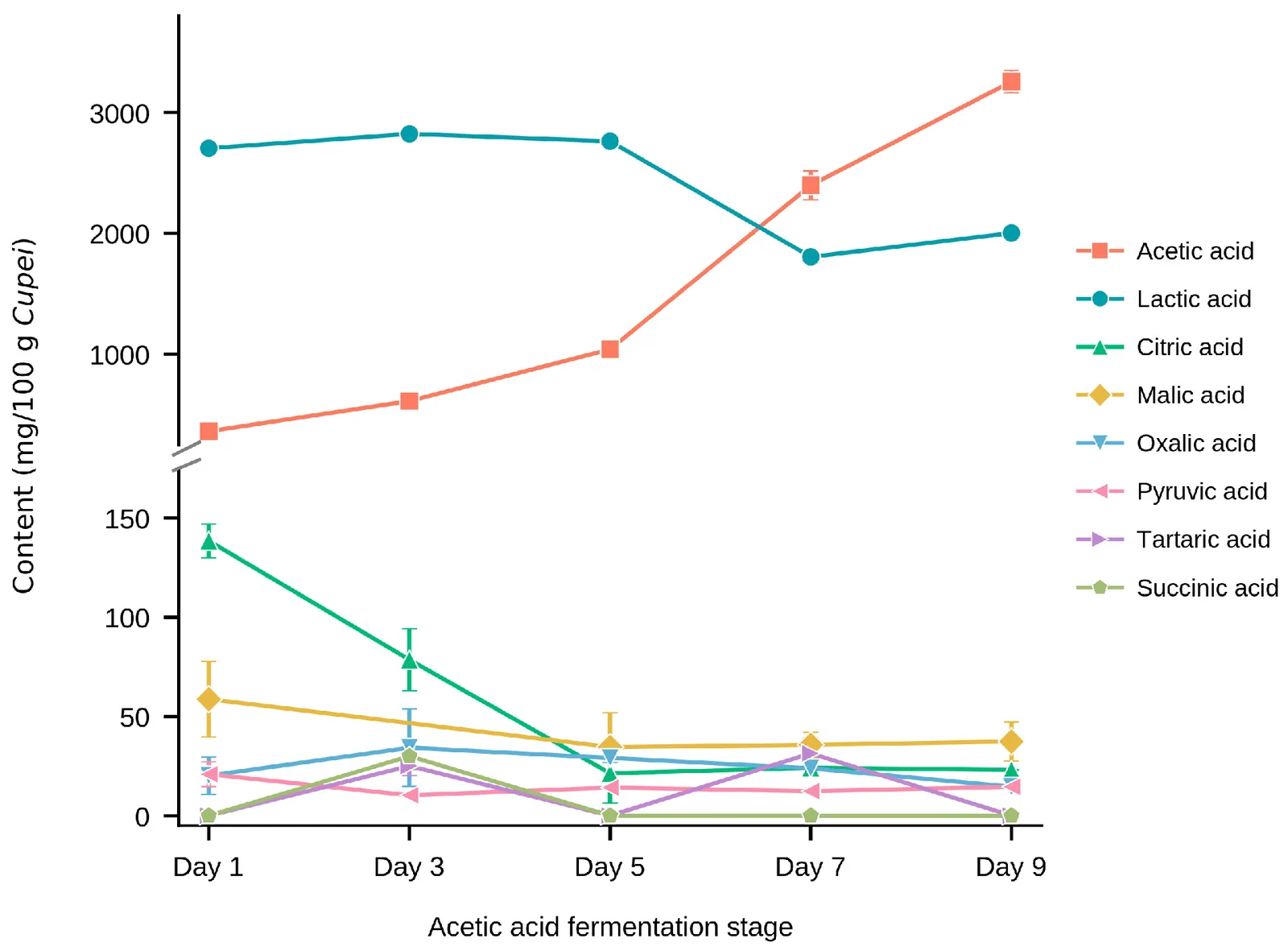
Dynamics of organic acids during acetic acid fermentation (AAF) of Shanxi aged vinegar (SAV).

The dynamics of the major organic acids across the AAF period (days 1–9) are shown in Figure 1. Lactic acid and acetic acid were the predominant organic acids, together accounting for more than 85% of the total identified organic acids. Lactic acid content first increased from 2704.5 ± 21.9 mg/100 g *Cupei* on day 1 to a maximum of 2823.4 ± 0.7 mg/100 g *Cupei* on day 3, and then progressively decreased to approximately 1800–2000 mg/100 g *Cupei* by the end of fermentation. In contrast, acetic acid accumulated steadily throughout the fermentation, rising from 361.7 ± 27.8 mg/100 g *Cupei* on day 1 to 3255.9 ± 92.8 mg/100 g *Cupei* on day 9. Total organic acids increased continuously, reaching 5348.2 ± 157.5 mg/100 g *Cupei* at the end of fermentation. This temporal transition from lactic acid accumulation in the early phase to acetic acid dominance in the later phase reflects the succession of active microbial metabolic functions. Other organic acids detected, including tartaric, oxalic, succinic, citric, pyruvic, and malic acids, remained at low levels throughout the fermentation. The formation of these organic acids is coupled to the microbial carbon metabolism, in which sugars and ethanol derived from the fermentation substrates serve as the primary carbon sources driving the flux toward organic acid production.

### 3.2. Community Structure by Integrated Meta-Omics

**Figure 2:**
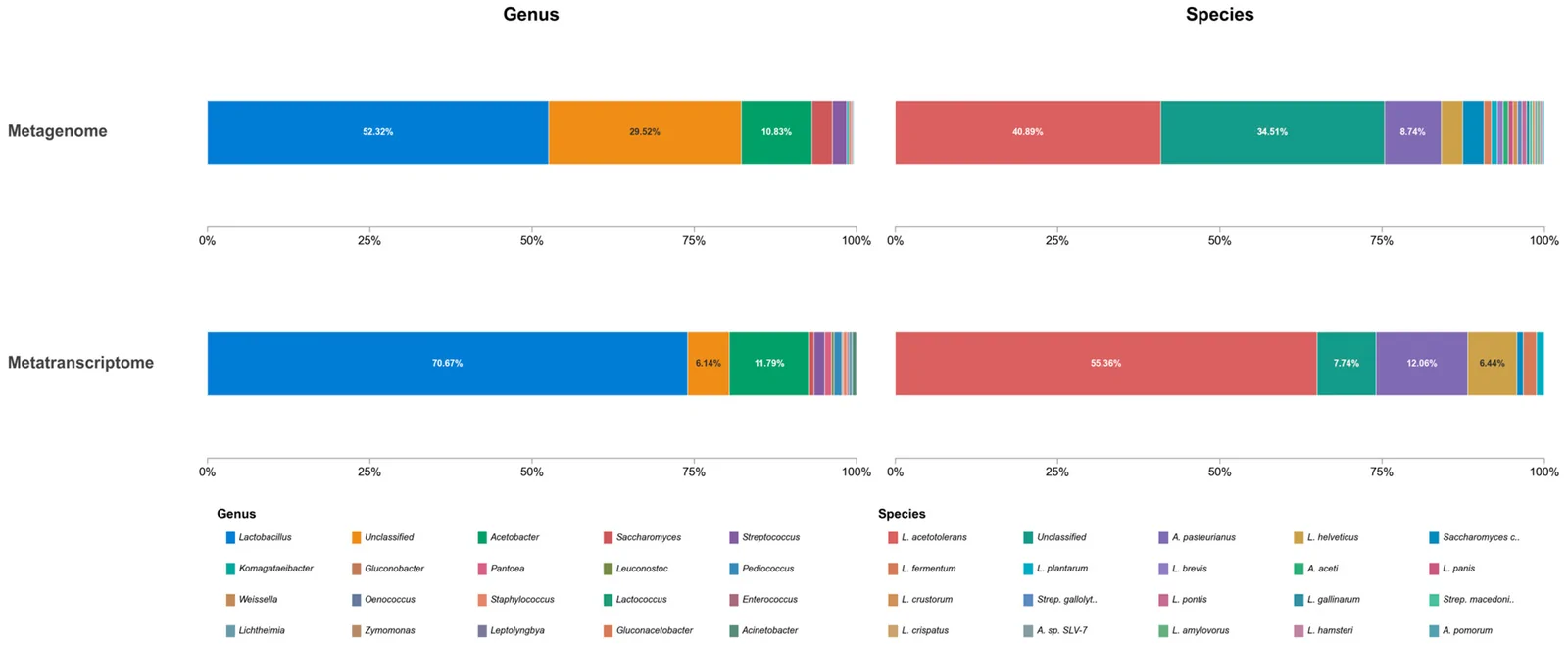
Microbial community composition revealed by metagenomic and metatranscriptomic sequencing.

Metagenomic sequencing identified 186 genera and 644 species, while metatranscriptomics detected 818 genera and 1786 transcriptionally active species (Figure 2). Figure 2 presents the top 20 genera and species detected by metagenomic and metatranscriptomic sequencing of SAV *Cupei*. At the genus level, *Lactobacillus* dominated both datasets (52.46% of genomic abundance; 70.67% of transcriptomic abundance), followed by *Acetobacter* (10.86%; 11.79%). *Saccharomyces* (3.12% genomic), *Streptococcus* (2.22%), *Komagataeibacter* (0.35%), and *Gluconobacter* (0.27%) were also detected. Notably, *Komagataeibacter* and *Gluconacetobacter*, present at moderate genomic abundance, were absent from the transcriptionally active top 20, while *Bacillus* (1.44%), *Alternaria* (1.07%), and *Cryptococcus* (0.65%) showed significant transcriptional activity despite low genomic abundance.

At the species level (Figure 2), *L. acetotolerans* was overwhelmingly dominant (40.89% genomic; 55.36% transcriptional), followed by *A. pasteurianus* (8.74%; 12.06%) and *Lactobacillus helveticus* (3.31%; 6.44%). Additional species including *Lactobacillus fermentum* (1.14%), *Lactobacillus plantarum* (0.92%), *Lactobacillus brevis* (0.90%), *Acetobacter aceti* (0.82%), *Lactobacillus casei* (0.71%). and *P. acidilactici* was also detected at lower abundances, accounting for only 0.11% of the total species-level abundance. The top three most abundant species were consistent between the two datasets, further confirming the dominant roles of *Lactobacillus* and *Acetobacter* in the SAV ecosystem.

### 3.3. Source Tracking of Organic Acid Synthesis Genes

**Figure 3:**
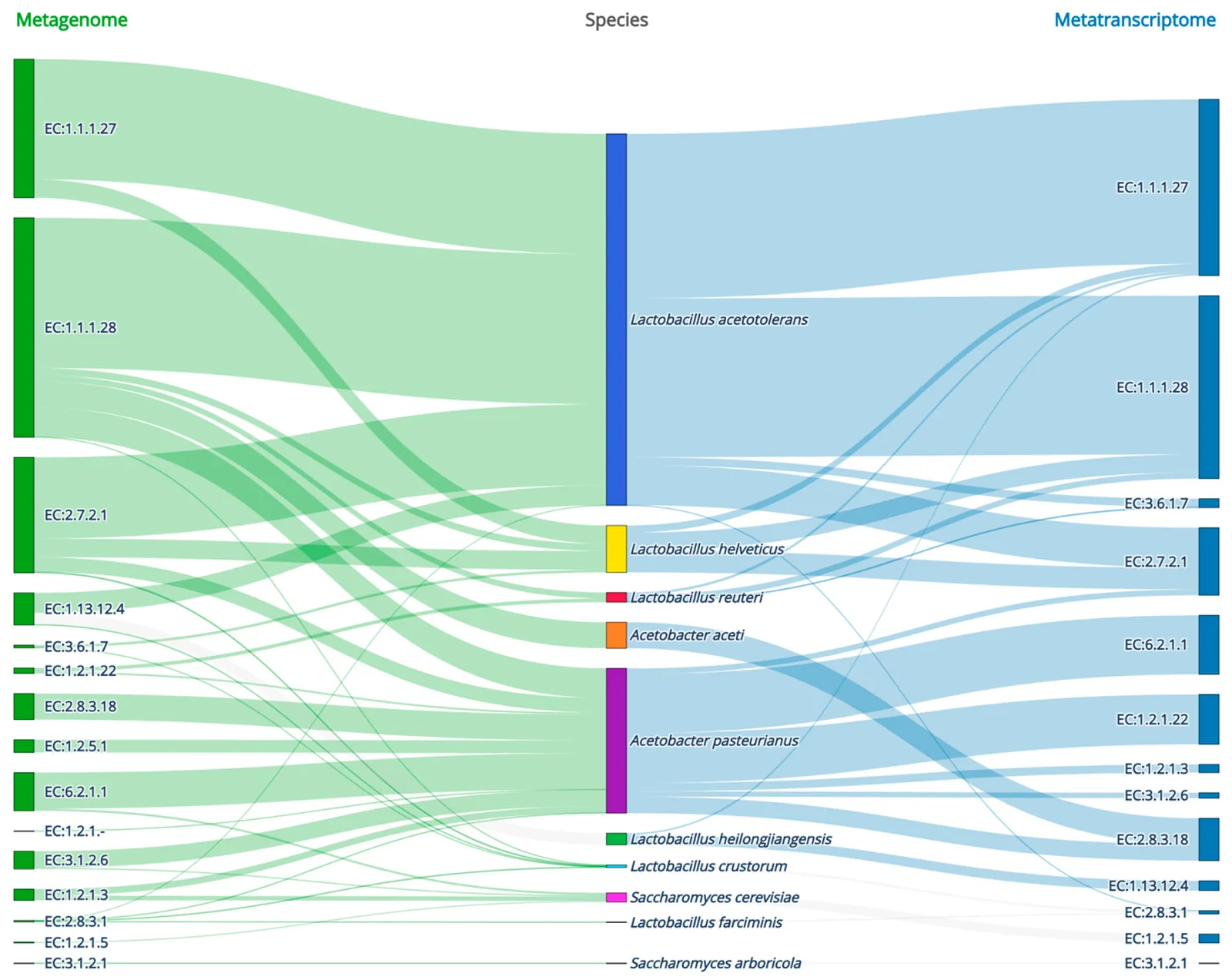
Source tracking of organic acid metabolism-associated EC categories across core microbial species in Shanxi aged vinegar fermentation.

The Sankey diagram (Figure 3) visualizes the distribution of 10 core microbial species across 12 EC functional categories involved in acetate and lactate metabolism, comparing metagenomic coding potential (left, green flow) with metatranscriptomic expression (right, blue flow). Each species’ total flow width was normalized by a shared weight factor to eliminate visual bias from unilateral data absence, enabling simultaneous comparison of inter-species abundance differences and intra-species functional structures.

*L. acetotolerans* displayed the widest total flow, several-fold wider than all other species combined, confirming its ecosystem dominance. In the source-tracking analysis, the genomic and transcriptomic flow of EC:1.1.1.27 (L-lactate dehydrogenase) and EC:1.1.1.28 (D-lactate dehydrogenase) was predominantly attributed to *L. acetotolerans* (approximately 70–87%), indicating that *L. acetotolerans* is the major contributor of lactate dehydrogenase genes in the community. Notably, EC:1.13.12.4 (lactate 2-monooxygenase) comprised 5.4% at the genomic level but was completely undetectable transcriptionally, suggesting this gene is silenced under the prevailing conditions. EC:3.6.1.7 (acylphosphatase) showed the opposite pattern: absent in the genome but detected at low transcriptional levels, possibly reflecting assembly or annotation differences.

*A. pasteurianus* exhibited the most complex EC profile (10 EC categories), and its genome–transcriptome discordance was particularly striking. EC:1.2.1.22 (lactaldehyde dehydrogenase) comprised only 1.3% of the genomic coding potential but surged to 34.5% of transcriptional activity—a 26-fold enrichment—suggesting a critical role in current metabolic activity. By contrast, EC:1.1.1.28 constituted 20.5% of the genome but showed zero transcriptional activity. EC:6.2.1.1 (acetyl-CoA synthetase) and EC:2.8.3.18 (succinyl-CoA:acetate CoA-transferase) maintained high levels in both datasets, indicating constitutively active carbon metabolism pathways.

Four species (*Lactobacillus crustorum*, *Lactobacillus farciminis*, *Saccharomyces cerevisiae*, and *Saccharomyces arboricola*) showed zero transcriptional activity (grey fill), indicating either dormancy/low metabolic state or roles as a genetic reserve rather than active functional contributors. EC:1.1.1.27 and EC:1.1.1.28 formed a “core functional module” conserved across multiple *Lactobacillus* species, whereas EC:2.8.3.18 and EC:6.2.1.1 were biased toward *Acetobacter*, reflecting the metabolic division of labor between AAB and LAB.

### 3.4. Metabolic Interaction Network Reveals Co-Inhibition of P. acidilactici

Although metagenomic and metatranscriptomic data consistently identified *L. acetotolerans* as the most abundant and transcriptionally active species, our previous culture-dependent observations suggested that this strain may have limited culturability and acid-producing performance under laboratory conditions [34]. In contrast, *P. acidilactici* AAF1-5, despite its low abundance, demonstrated excellent thermotolerance and lactic acid production capability [29]. To understand this “high-abundance, low-performance” paradox and investigate the ecological constraints on *P. acidilactici* within the vinegar microbiota, a metabolic interaction network of the SAV *Cupei* microbial community was constructed, visualizing the unidirectional metabolic influences exerted by the two hub strains—*A. pasteurianus* and *L. acetotolerans*—on other community members. The network comprises 50 microbial species, including the 2 hub strains, *P. acidilactici*, other Acetobacteraceae, LAB, and non-LAB species, with edges representing significant metabolic interactions.

**Figure 4:**
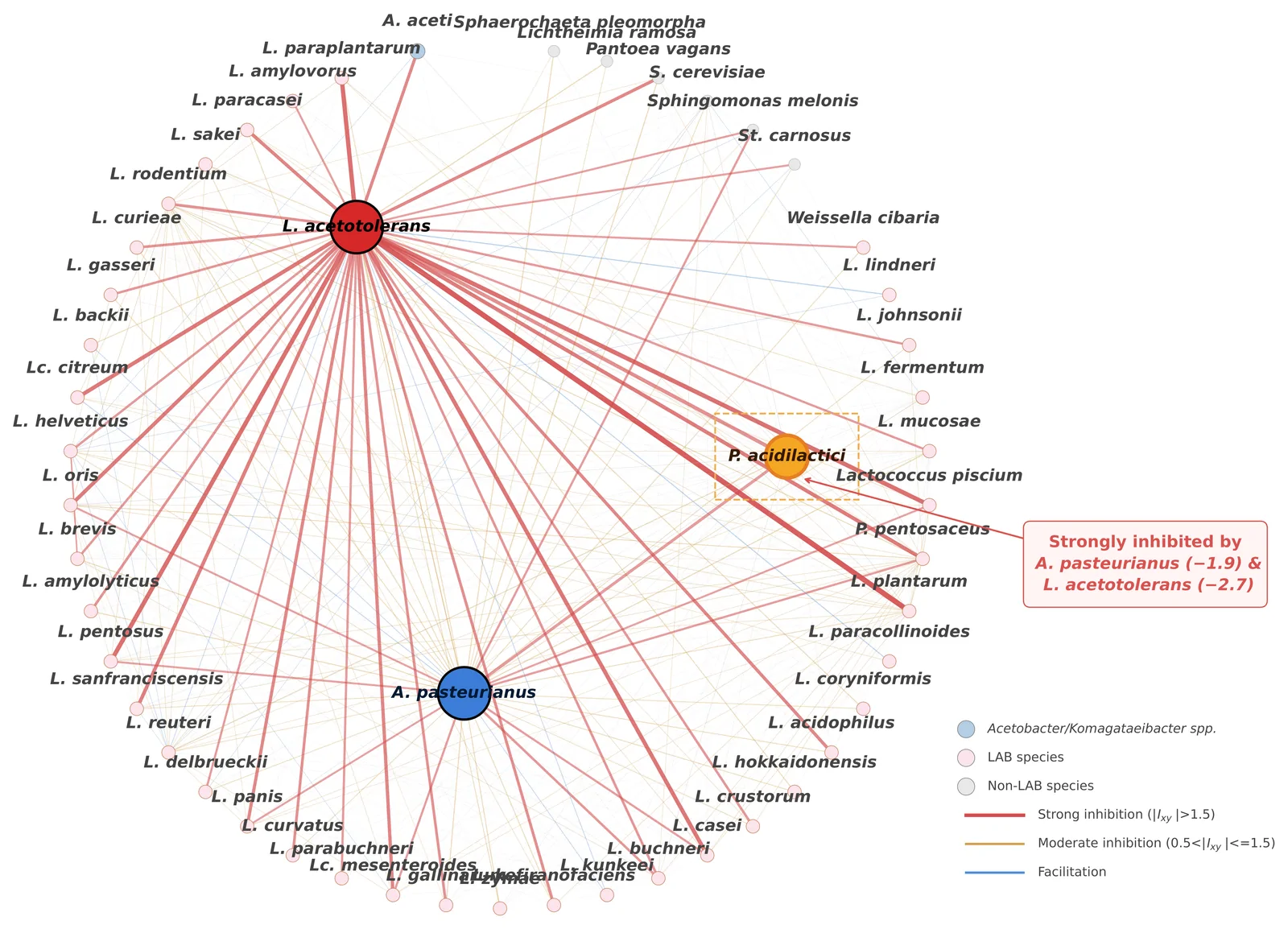
Directed metabolic interaction network (*I_xy_*) centered on *Acetobacter pasteurianus* and *Lactobacillus acetotolerans* in the Shanxi aged vinegar (SAV) microbial community. The network depicts unidirectional metabolic influences exerted by the two dominant species, *A. pasteurianus* (blue node) and *L. acetotolerans* (red node), on other microbial members, with only significant interactions shown (*|I_xy_|* > 0.3). *Pediococcus acidilactici* (gold node) is highlighted as the target strain. Edge thickness reflects the magnitude of the *I_xy_* value. Edges were classified as “strong” or “moderate” inhibition and “facilitation” according to the *I_xy_* cutoffs described in Section 2.4. Note that *P. acidilactici* was identified as the best chassis candidate on the basis of its optimal growth and acid production in the strain screening.

The network revealed that *A. pasteurianus* and *L. acetotolerans* constitute two central hub nodes, exerting pronounced metabolic effects on multiple community members (Figure 4). *L. acetotolerans* displayed pervasive negative metabolic interactions toward other LAB species, with particularly strong inhibition of *L. helveticus* (*I_xy_* = −2.128), *L. casei* (*I_xy_* = −4.555), and *P. acidilactici* (*I_xy_* = −2.737). *A. pasteurianus* similarly exerted negative influences on all LAB members, with an interaction force of *I_xy_* = −1.887 toward *P. acidilactici*. Notably, *P. acidilactici* was subject to significant co-inhibition from both core species (mean *I_xy_* = −2.312), suggesting that its limited abundance in the natural microbiota may be attributed to ecological suppression by the two dominant hub strains. Consistently, its lactate dehydrogenase genes (EC:1.1.1.27 and EC:1.1.1.28) showed negligible transcriptional activity in the metatranscriptomic data (Figure 3); this is consistent with the strain being suppressed in its native niche, and its lactic acid production capacity was not realized in situ.

Taken together with the excellent pure-culture performance of *P. acidilactici* AAF1-5, these network results suggest that its low abundance in the SAV *Cupei* ecosystem may be associated with ecological niche competition. Wu et al. [5] reported that *L. acetotolerans* was the most abundant LAB during early AAF and its high abundance gave it a competitive advantage in carbon source utilization, yet the expression of its lactate dehydrogenase genes (*ldhA* and *ldh*) was significantly inhibited under high-temperature conditions. This provides indirect support for the view that the suppressive effect of *L. acetotolerans* on *P. acidilactici* (*I_xy_* = −2.737) may be driven by resource competition rather than superior metabolic activity. Meanwhile, *A. pasteurianus* has been confirmed as the key producer of acetic acid throughout the AAF process [25], and its sustained acid production creates a progressively hostile environment for acid-sensitive microorganisms, consistent with its negative interaction force toward *P. acidilactici* (*I_xy_* = −1.887). Therefore, we infer that engineering the acid tolerance of *P. acidilactici* could relieve its ecological constraints in the native environment, potentially unlocking its functional potential.

### 3.5. Construction and Characterization of Acid-Tolerant Recombinant P. acidilactici

**Figure 5:**
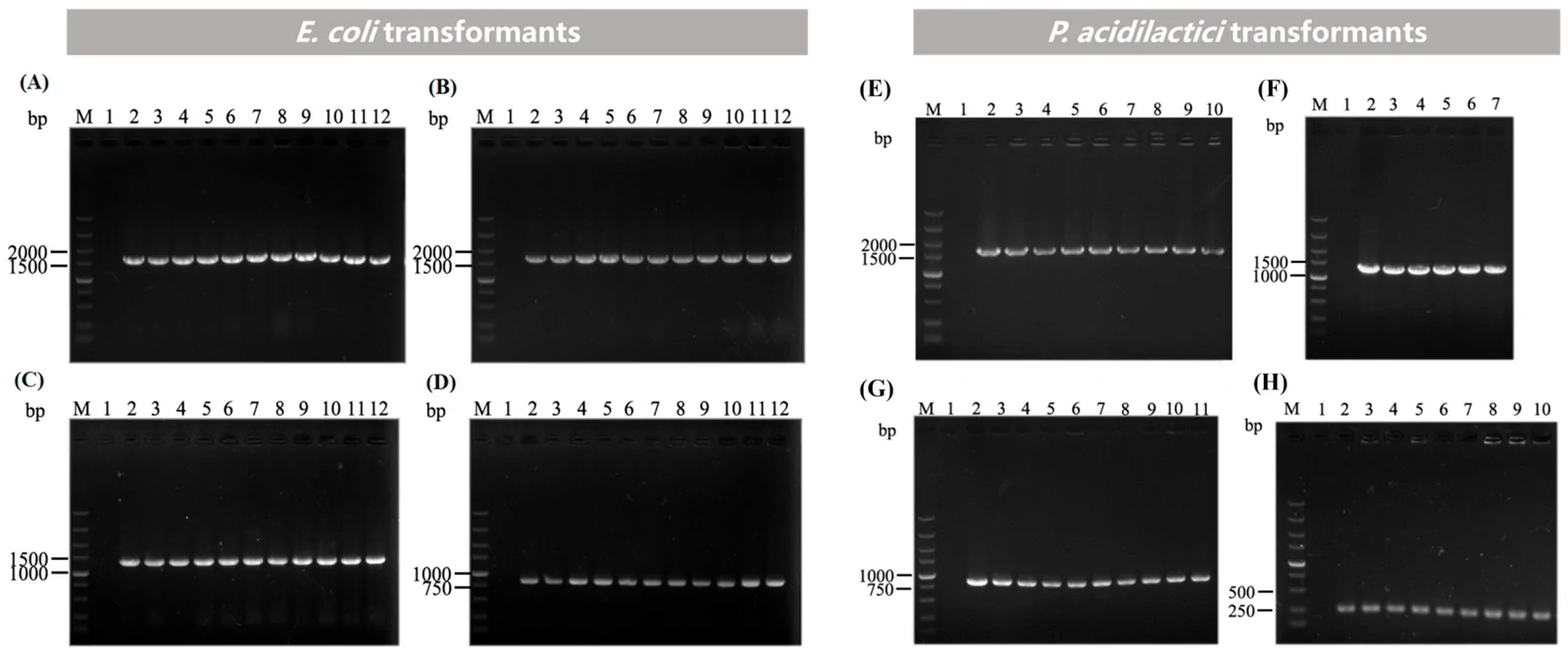
Construction and PCR verification of recombinant *Pediococcus acidilactici* strains. (**A**) pNZ8148-*adhA* transformants (approximately 1598 bp); (**B**) *pNZ8148-adhB* transformants (approximately 1643 bp); (**C**) pNZ8148-*aatA* transformants (approximately 1304 bp); and (**D**) pNZ8148-*grpE* transformants (approximately 821 bp). (**E**–**H**) Colony PCR verification of plasmid transformation into *P. acidilactici* AAF1-5. (**E**) pNZ8148-*adhB*; (**F**) pNZ8148-*aatA*; (**G**) pNZ8148-*grpE*; and (**H**) empty vector control (pNZ8148). M, DL5000 DNA marker; N, negative control (wild-type strain); numbered lanes indicate individual transformant colonies.

The acid-resistance genes *adhA* (2229 bp), *adhB* (1419 bp), *aatA* (1809 bp), and *grpE* (597 bp) were successfully amplified from *A. pasteurianus* AC2005 and cloned into pNZ8148 (Figure S1) (Figure 5A–D). Sequencing confirmed 100% amino acid identity for *adhA*, *adhB*, and *grpE* and 99.67% for *aatA*. The recombinant plasmids were subsequently electrotransformed into *P. acidilactici* AAF1-5. Colony PCR verification confirmed successful introduction of pNZ8148-*adhB*, pNZ8148-*aatA*, and pNZ8148-*grpE* into *P. acidilactici* AAF1-5 (Figure 5E–G), generating the recombinant strains *P. acidilactici*-*adhB*, *P. acidilactici*-*aatA*, and *P. acidilactici*-*grpE*. The *adhA* recombinant strain was not obtained reproducibly and was therefore excluded from subsequent functional analyses; because adhA and adhB are functionally redundant members of the same Adh family, successful expression of either one is sufficient to represent this resistance mechanism.

**Figure 6:**
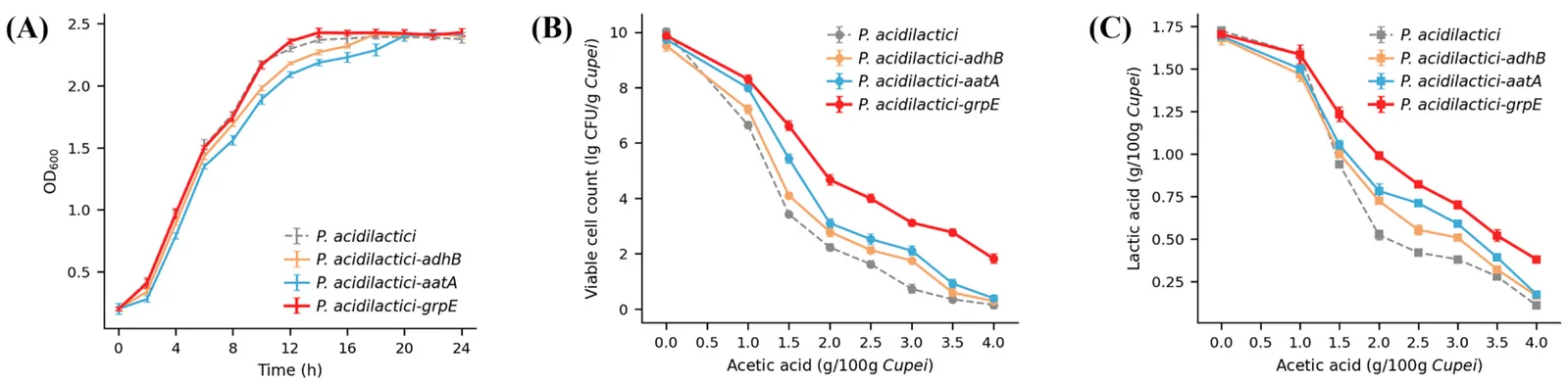
Growth performance and acid tolerance of *Pediococcus acidilactici* AAF1-5 and recombinant strains under acetic acid stress. (**A**) Growth curves of *P. acidilactici* and recombinant strains (*P. acidilactici*-*adhB*, *P. acidilactici*-*aatA*, and *P. acidilactici*-*grpE*) during cultivation; (**B**) Viable cell counts of the parental and recombinant strains exposed to different concentrations of acetic acid; (**C**) Lactic acid production by the parental and recombinant strains under acetic acid stress.

All recombinant strains reached the stationary phase within 14–20 h. *P. acidilactici*-*grpE* most closely resembled the wild-type growth pattern but achieved a 15% higher peak biomass (Figure 6A). Under acetic acid stress (Figure 6B,C), the viability of wild-type *P. acidilactici* fell below 10^4^ CFU/g *Cupei* at acetic acid concentrations exceeding 1 g/100 g *Cupei*, and lactic acid production dropped below 1.0 g/100 g *Cupei*. All three recombinant strains showed improved tolerance. At 1.5 g/100 g *Cupei*, the viable counts of *P. acidilactici*-*adhb*, *P. acidilactici*-*aata*, and *P. acidilactici*-*grpE* reached 1.3 × 10^4^, 2.7 × 10^5^, and 4.2 × 10^6^ CFU/g *Cupei*, respectively, and their lactic acid yields were 1.00, 1.05, and 1.23 g/100 g *Cupei*, with *P. acidilactici*-*grpE* showing the highest production. At 4 g/100 g *Cupei*, *P. acidilactici*-*grpE* maintained viable counts of approximately 66 CFU/g *Cupei* and produced 0.38 g/100 g *Cupei* lactic acid, representing approximately 47-fold and 3.5-fold improvements over the wild-type strain, respectively.

### 3.6. Simulated Fermentation Validation of P. acidilactici-grpE

To investigate the regulatory effect of the recombinant *P. acidilactici*-*grpE* strain on lactic acid metabolism in the vinegar fermentation system, SSSF was carried out using freshly discharged SAV *Cupei*. Prior to simulated fermentation validation, the basic fermentation parameters of the wild-type and recombinant strains were compared under laboratory liquid pure-culture conditions to assess whether the heterologous expression of *grpE* affected general fermentation performance. As shown in Table 1, there were no significant differences between *P. acidilactici* AAF1-5 and *P. acidilactici*-*grpE* in pH, residual sugar, total acid, lactic acid, and acetic acid contents (*p* > 0.05), confirming that the molecular manipulation did not compromise the strain’s basic fermentation properties.

**Figure 7:**
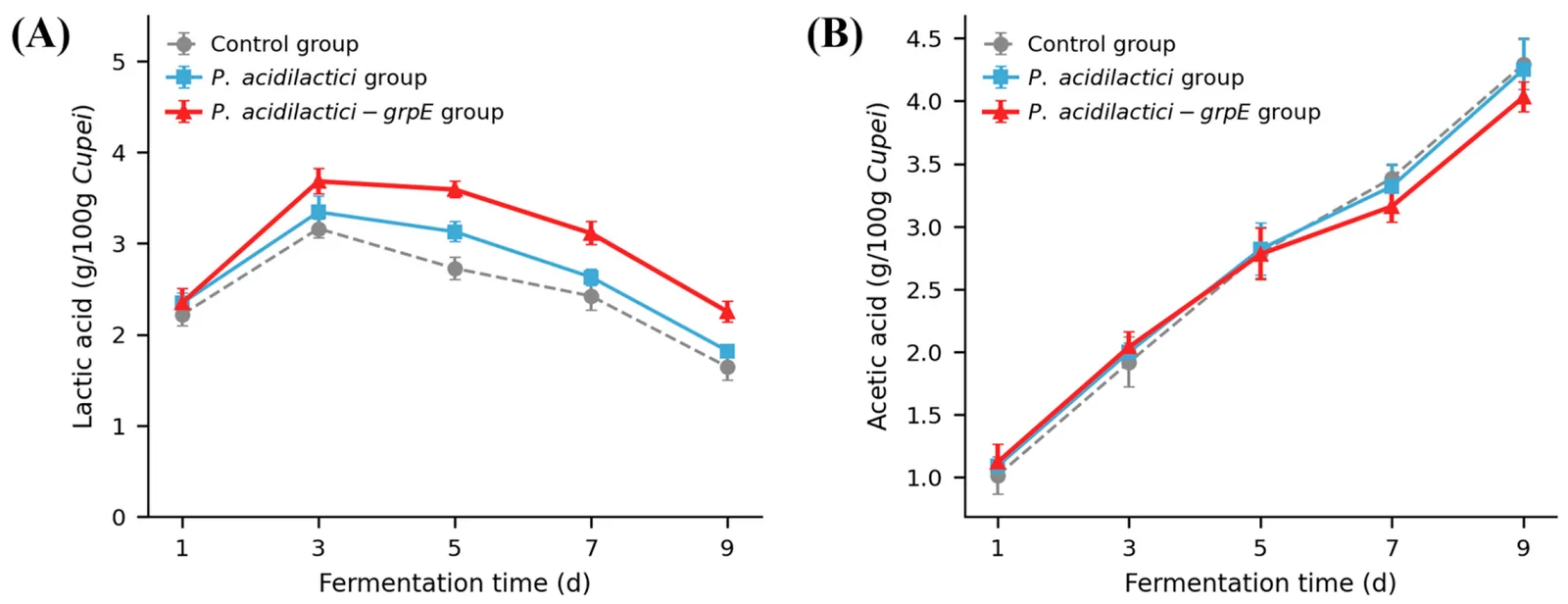
Changes in lactic acid and acetic acid contents during fermentation in simulated vinegar brewing. (**A**) Lactic acid production by the control group, *Pediococcus acidilactici* AAF1-5, and *P. acidilactici*-*grpE *strains during fermentation; (**B**) Acetic acid accumulation in the simulated vinegar brewing system inoculated with different strains.

To further evaluate the regulatory effect of the recombinant strain on lactic acid production in the vinegar brewing system, SSSF was conducted using freshly discharged *Cupei* (day 1 of SAV AAF). The dynamics of lactic acid and acetic acid throughout the fermentation are shown in Figure 7. Lactic acid content in all groups exhibited an initial increase followed by a decline, and the addition of both *P. acidilactici* and *P. acidilactici*-*grpE* enhanced lactic acid levels compared with the blank control. The *P. acidilactici*-*grpE* group consistently showed the highest lactic acid levels throughout the fermentation. At the end of fermentation, the lactic acid content in the *P. acidilactici*-*grpE* group reached 2.25 g/100 g *Cupei*, representing a 23.63% increase over the wild-type group (1.82 g/100 g *Cupei*) and a 37.20% increase over the blank control (1.64 g/100 g *Cupei*). No significant differences in acetic acid content were observed among the three groups during the early and middle stages of fermentation. However, in the later stage, the *P. acidilactici*-*grpE* group exhibited a slightly lower acetic acid content, likely due to the utilization of acetic acid as a carbon source by *P. acidilactici* under carbon-limited conditions [35]. At the end of fermentation, the lactic acid/acetic acid ratio in the *P. acidilactici*-*grpE* group was elevated by 47.37% compared with the wild-type group and by 30.23% compared with the blank control. These results demonstrate that heterologous expression of the acid-resistance gene *grpE* from *A. pasteurianus* in *P. acidilactici* AAF1-5 can effectively enhance lactic acid content and improve the lactic acid/acetic acid ratio at the end of SAV acetic acid fermentation.

## 4. Discussion

A central finding of this study is the decoupling between ecological abundance and physiological performance in the SAV microbial community. Although *L. acetotolerans* dominated both the metagenome and metatranscriptome, *P. acidilactici*—a member of the rare biosphere—exhibited superior physiological performance under fermentation-relevant conditions. This decoupling between ecological abundance and cultivation-based physiological performance challenges the widely held assumption that the most abundant taxa identified by sequencing are necessarily the most functionally relevant [25,26]. It indicates that ecological dominance at the population level does not necessarily translate into superior metabolic capacity at the cellular level.

Several factors at different levels may explain this decoupling. At the methodological level, metagenomics and metatranscriptomics measure gene and transcript abundance, respectively, but neither directly measures per-cell metabolic flux. A species can maintain high transcript levels for housekeeping functions and stress survival without committing high enzymatic flux to lactate biosynthesis [36]. Notably, when normalized to *L. acetotolerans*’ own transcriptome, the lactate dehydrogenase genes (EC:1.1.1.27 and EC:1.1.1.28) accounted for only ~0.4% of its total transcriptional output, whereas its transcription was dominated by housekeeping and energy-metabolism functions, including glycolysis (EC:1.2.1.12, ~11.5%), ATP synthase (EC:3.6.3.54, ~7.8%), and nucleotide metabolism (EC:2.7.7.6, ~6.2%). At the ecosystem level, the SSF environment imposes multifactorial stress—high temperature (up to 47 °C), elevated acidity, and ethanol—that intensifies as fermentation progresses, selecting for robust generalists with broad stress tolerance rather than narrow specialists [7]. At the biological level, *L. acetotolerans* is itself a metabolically narrow specialist with a limited carbohydrate utilization spectrum [37]; its high abundance in early AAF likely reflects rapid ecological niche preemption at the onset of fermentation rather than sustained high metabolic throughput [38]. Together, these observations suggest that phenotype-level trait integration—the capacity to withstand multiple simultaneous stresses and sustain high metabolic output—is poorly predicted by sequence-based abundance or transcriptional data alone.

The present findings connect to the broader ecological concept of the rare biosphere [20]. Studies on *Daqu* fermentation [21,24], vinegar SSF [22], and cheese [14] have demonstrated that low-abundance community members can play functionally important roles. However, these earlier studies were largely descriptive or correlative, and the question of why these organisms remain rare in their native ecosystems has received less attention. The present study goes beyond this by (i) quantitatively identifying the co-inhibitory pressures acting on *P. acidilactici* through a species-resolved metabolic interaction network, which suggested that this rare biosphere member being subject to co-inhibition by both *L. acetotolerans* (*I_xy_* = −2.737) and *A. pasteurianus* (*I_xy_* = −1.887) and (ii) experimentally relieving one of the identified constraints (acid stress) through a targeted genetic intervention and demonstrating a functional consequence in a simulated fermentation. Given that *P. acidilactici* demonstrates superior physiological performance in pure culture, its low abundance in situ therefore appears to reflect ecological constraints identified in the network analysis, of which only the acid-stress component was experimentally tested here. This indicates that low abundance should not be interpreted as low functional potential.

To experimentally test this ecological hypothesis—that environmental constraints, not intrinsic metabolism, limit *P. acidilactici* in the SAV ecosystem—we selected one of the identified constraints, acid stress, and attempted to relieve it. The acid-resistance gene *grpE* from *A. pasteurianus* was heterologously expressed in *P. acidilactici* AAF1-5 as an experimental tool, rather than as an industrial optimization strategy. *grpE* functions as a nucleotide-exchange factor for the DnaK-DnaJ chaperone system, facilitating protein refolding under acid stress [12,39]. If the ecological hypothesis were correct—that *P. acidilactici* possesses intrinsic metabolic capacity that is masked by environmental stress—then enhancing its acid tolerance should allow it to express its functional potential. The results support this hypothesis: engineering acid tolerance by heterologous expression of *grpE* substantially improved both survival and lactate production under fermentation-relevant conditions. It should be noted that this experiment specifically supports the acid-stress arm of the ecological constraint hypothesis, whereas resource competition with *L. acetotolerans* remains an inference from the network analysis and has not been experimentally validated in the present study. Directly testing the resource-competition arm, for example through co-culture competition assays between *P. acidilactici* and *L. acetotolerans* (and/or the other dominant LAB), is a priority for future work. Collectively, these findings support the acid-stress arm of our original hypothesis: the low abundance of *P. acidilactici* is not attributable to insufficient intrinsic physiological capacity. The contribution of resource competition, by contrast, remains to be tested experimentally.

Despite these findings, several limitations remain. First, the simulated fermentation system does not fully recapitulate the industrial SAV process, and scaled validation under authentic production conditions is necessary. Second, the molecular targets of *grpE*-mediated protection require proteomic elucidation. Finally, food safety assessment, including plasmid stability, marker removal, and food-grade expression system development, is essential before any practical application. Addressing these questions will further clarify how ecological constraints shape microbial function in complex fermentation ecosystems.

## 5. Conclusions

This study demonstrates that meta-omic abundance should not be directly interpreted as functional importance in the SAV microbial community. The decoupling between ecological abundance and physiological performance—in which the most abundant species (*L. acetotolerans*, 40.89%) proved physiologically inferior to a rare biosphere member (*P. acidilactici*, 0.11%)—is consistent with the view that ecological interactions, particularly competition and acid stress, can obscure the intrinsic physiological potential of low-abundance microorganisms. By integrating community profiling, interaction network analysis, and physiological characterization, this study demonstrated that *P. acidilactici* possesses latent functional capacity that is masked, at least in part, by acid stress in its native niche. Heterologous expression of *grpE*, used as an experimental tool to test this ecological hypothesis, lends support to the notion that relieving a specific environmental constraint (acid stress) allowed this rare-biosphere member to express its functional potential. It should be emphasized that this experimental validation specifically addresses the acid-stress constraint, whereas resource competition remains to be investigated for future experimental testing. This study therefore provides an ecological framework for interpreting abundance–function relationships in complex fermentation microbiomes. These findings highlight the importance of integrating ecological context with meta-omic analyses when identifying functionally relevant microorganisms in complex microbial ecosystems. From a practical perspective, identifying ecologically constrained yet functionally potent rare taxa—together with strategies to relieve specific constraints such as acid stress—may inform starter-culture design or bioaugmentation in SAV production, pending food-safety assessment and industrial-scale validation.

## Figures and Tables

**Table 1:** The comparison of the fermentation performance of original strain (*P. acidilactici*) and the recombinant strain (*P. acidilactici*-*grpE*) under laboratory liquid pure-culture conditions.

Strains	pH	Residual Sugar	Total Acid	Lactic Acid	Acetic Acid
		(g/100 g *Cupei*)			
*P. acidilactici*	3.51 ± 0.01 ^a^	1.15 ± 0.03 ^a^	2.66 ± 0.04 ^a^	1.71 ± 0.03 ^a^	0.26 ± 0.02 ^a^
*P. acidilactici*-*grpE*	3.54 ± 0.05 ^a^	1.21 ± 0.04 ^a^	2.63 ± 0.04 ^a^	1.68 ± 0.04 ^a^	0.22 ± 0.02 ^a^

Note: The same letters indicate no significant differences (*p* > 0.05).

## Data Availability

The data presented in this study are openly available in NCBI Short Read Archive at https://www.ncbi.nlm.nih.gov/sra (accessed on 18 August 2026), reference numbers PRJNA689964 and PRJNA602043.

## Supplementary Materials

The following supporting information can be downloaded at: https://www.mdpi.com/article/10.3390/foods15162942/s1, Table S1 Sequences of primers; Figure S1 Agarose gel electrophoresis of PCR-amplified target genes and restriction enzyme-digested plasmid pNZ8148.
