# Supplementary material for: Rare Biosphere Reveals a Decoupling Between Microbial Abundance and Intrinsic Physiological Potential in Shanxi Aged Vinegar Fermentation
Source: Foods. 2026 Aug 21;15(16):2942. doi: 10.3390/foods15162942 (PMC13512557; doi:10.3390/foods15162942)
Supplement: Supplementary file 1 [file foods-15-02942-s001.zip › foods-4488689-supplementary.pdf]

Table S1 Sequences of primers

Primer Name	Sequence (5’-3’)
adhA-F	gtactgcagctcgagggtaccATGACCCGCCCCGCCTCCGCCAAGA
adhA-R	agaaagcttgagctctctagaTTAGGGGTTAATGCCAAGTGTCGGGT
adhAyz1-R	TGCAGATCCTTGATGTAAGCGGTAC
adhB-F	gtactgcagctcgagggtaccATGATGATTAACAGGCTAAAAGCTGC
adhB-R	agaaagcttgagctctctagaTTACTGTGCTTCATCCACACCAGCATGT
grpE-F	gtactgcagctcgagggtaccATGACCCAAGATACAGATCCCACAC
grpE-R	agaaagcttgagctctctagaTTAGGAAGCCTTGGCAACCACC
aatA-F	gtactgcagctcgagggtaccATGGCGCATCCTCCTTTACTCCAC
aatA-R	agaaagcttgagctctctagaTTAGTCTGCCTGCAGAGCCTCGC
aatAyz-F	GCCTGGCGTGAAGAGGTGCTGGAA
8148-F	GATAACGCGAGCATAATAAACG
8148-R	TTGGCTATCAATCAAAGCAACACG

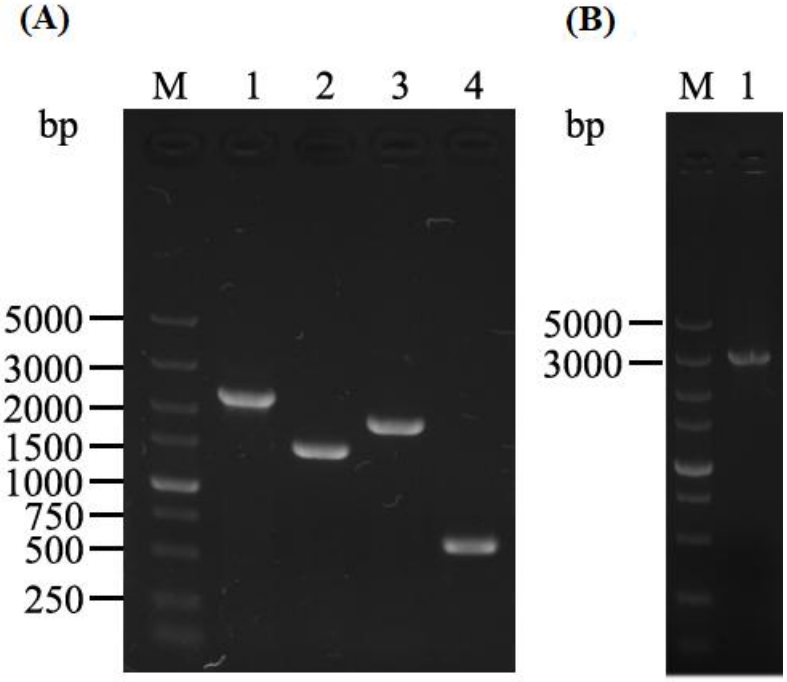

Figure S1 Agarose gel electrophoresis of PCR-amplified target genes and restriction enzyme-digested plasmid pNZ8148. (A) PCR products of *adhA*, *adhB*, *aatA*, and *grpE* (lanes 1-4, respectively), showing specific bands of approximately 2229 bp, 1419 bp, 1809 bp, and 597 bp, consistent with the expected sizes. (B) Double digestion of plasmid pNZ8148 with *Xba*I and *Kpn*I, yielding a specific band of approximately 3167 bp, consistent with the linearized plasmid. M, DL5000 bp DNA marker.
